# Determinants of Prolonged Postoperative Length of Stay After Carotid Revascularization: A Single-Center Registry Analysis from Kazakhstan

**DOI:** 10.3390/jcm15176548

**Published:** 2026-08-25

**Authors:** Almas Shamshiyev, Shokan Kaniyev, Askar Matkerimov, Manat Zhakubayev, Talgat Demeuov, Mead Khanchi, Almas Saduakas, Rustam Makkamov, Nurlybek Yerkinbayev, Alisher Kozhamkul, Gulnaz Nurlybaeva, Mukhtar Kulimbet

**Affiliations:** 1 Department of Vascular Surgery, Syzganov National Scientific Center of Surgery, Almaty 050004, Kazakhstan; almas.shamshiyev@gmail.com (A.S.); askar151282@gmail.com (A.M.); mjakubayev@gmail.com (M.Z.); talgadem@mail.ru (T.D.); khanchi.mead@yahoo.com (M.K.); makkamovrustam7@gmail.com (R.M.); nurlybekerkinbaev@gmail.com (N.Y.); alisherkozhamkul@gmail.com (A.K.); 2 Department of Hepatopancreatobiliary Surgery, Syzganov National Scientific Center of Surgery, Almaty 050004, Kazakhstan; shokan.kaniyev@gmail.com; 3 Department of Physical Medicine and Rehabilitation, Sports Medicine, Asfendiyarov Kazakh National Medical University, Almaty 050026, Kazakhstan; 4 Research Management Department, Research Institute of Cardiology and Internal Diseases, Almaty 050000, Kazakhstan; mkbkul@gmail.com

**Keywords:** carotid stenosis, carotid endarterectomy, length of stay, postoperative complications, Kazakhstan

## Abstract

**Background/Objectives**: Carotid revascularization reduces stroke risk in patients with carotid stenosis. Postoperative length of stay (LOS) reflects resource utilization and recovery, yet its determinants remain poorly described in Central Asia. This study examined whether baseline patient characteristics predict prolonged postoperative LOS in a Kazakhstani referral cohort. **Methods**: We analyzed a single-center retrospective cohort (clinical registry) of 320 consecutive patients who underwent carotid revascularization between January 2018 and December 2025. Carotid endarterectomy (CEA) was performed in 88 patients (27.5%) and carotid artery stenting (CAS) in 228 (71.2%). Prolonged postoperative LOS was defined a priori as >8 days (75th percentile). Multivariable logistic regression was performed, with negative binomial regression; a model additionally including procedure type, and a model including in-hospital complications were used as sensitivity analyses. **Results**: Patients were predominantly male (74.7%) with a mean age of 71.1 ± 7.5 years and a high burden of comorbidities. Median postoperative LOS was 6 days (IQR 4–8), and 72 patients (22.5%) had prolonged LOS. Postoperative LOS was similar after endarterectomy and stenting (median 6 vs 5 days). In-hospital complications occurred in 11 patients (3.4%), with two deaths (0.6%). No baseline characteristic independently predicted prolonged LOS (all *p* > 0.05; AUC = 0.629; Hosmer–Lemeshow *p* = 0.45), and procedure type was not associated with prolonged LOS (adjusted OR 1.14, 95% CI 0.61–2.14). In contrast, in-hospital complications were strongly associated with prolonged LOS (adjusted OR 8.50, 95% CI 2.04–35.40; *p* = 0.003), although this estimate was imprecise owing to the small number of events. Median postoperative LOS increased from 6 days in patients without complications to 12.5 days in those with complications (*p* < 0.001). **Conclusions**: In this elderly, comorbidity-heavy cohort, prolonged postoperative LOS was associated with perioperative complications rather than baseline patient characteristics. Because complications lie on the causal pathway between baseline risk and hospital stay, this finding is best interpreted as hypothesis-generating: it suggests that efforts to shorten stay may be better directed toward complication prevention than preoperative risk stratification, a hypothesis that warrants prospective evaluation.

## 1. Introduction

Stroke remains one of the leading causes of death and acquired disability worldwide, as well as in Kazakhstan, and extracranial carotid artery stenosis is an important and treatable cause of ischemic stroke [1,2,3]. Carotid revascularization—by carotid endarterectomy or carotid artery stenting—reduces the long-term risk of ipsilateral stroke in appropriately selected patients, and current society guidelines position it as a cornerstone of stroke prevention in patients with significant stenosis [4,5]. Long-term trial data confirm that the two revascularization strategies achieve broadly comparable durable protection, so contemporary quality improvement increasingly focuses not on whether to revascularize but on how efficiently and safely the procedure is delivered [6,7].

Within that quality agenda, postoperative length of stay (LOS) has emerged as a practical, routinely captured indicator that links clinical recovery to resource utilization. Prolonged stay after carotid revascularization is associated with higher cost, exposure to nosocomial risk and constrained surgical throughput, and several North American analyses have sought to identify the patients most likely to stay longer. These studies generally implicate older age, symptomatic presentation, medical comorbidity, non-elective admission and postoperative complications as correlates of extended stay [8,9]. Hypertension control, active smoking and weekend timing of surgery have likewise been examined as modifiable contributors to perioperative outcome and stay [10,11,12,13].

All this evidence, however, derives from large administrative registries in high-income Western health systems with short baseline stays, and the determinants of LOS may not transfer to settings where admission pathways, comorbidity profiles and discharge practices differ substantially. Central Asian data are essentially absent from this literature, and it is unknown whether the baseline characteristics that predict prolonged stay in North American cohorts retain any predictive value in a referral population characterized by advanced age, very high cardiac comorbidity and longer customary hospitalization.

The health system in Kazakhstan differs in important ways from those that generated the existing evidence. In Kazakhstan and comparable post-Soviet settings, carotid revascularization is typically delivered within an inpatient pathway that includes several days of preoperative work-up, and discharge is frequently governed by administrative, rehabilitative and social considerations rather than by clinical readiness alone. These features produce a longer customary hospitalization than in North American or Western European short-stay models and may alter which factors drive variation in postoperative stay. Characterizing these determinants locally is a prerequisite for using LOS as a quality metric and for designing interventions that improve throughput without compromising safety.

We aimed to analyze a single-center carotid revascularization registry in Kazakhstan to determine whether routinely available baseline patient characteristics independently predict prolonged postoperative LOS, and to quantify the relative contribution of preoperative case-mix versus in-hospital events to variation in stay.

## 2. Materials and Methods

### 2.1. Study Design and Setting

We conducted a single-center, retrospective observational cohort study using a clinical registry of consecutive patients who underwent carotid revascularization for carotid artery stenosis at the Syzganov National Scientific Center of Surgery, a tertiary referral institution in Almaty, Kazakhstan, between January 2018 and December 2025. Data were abstracted from structured clinical records into a standardized database and cleaned before analysis. The study was reported in accordance with the Strengthening the Reporting of Observational Studies in Epidemiology (STROBE) statement for cohort studies [14].

### 2.2. Participants

**Figure 1:**
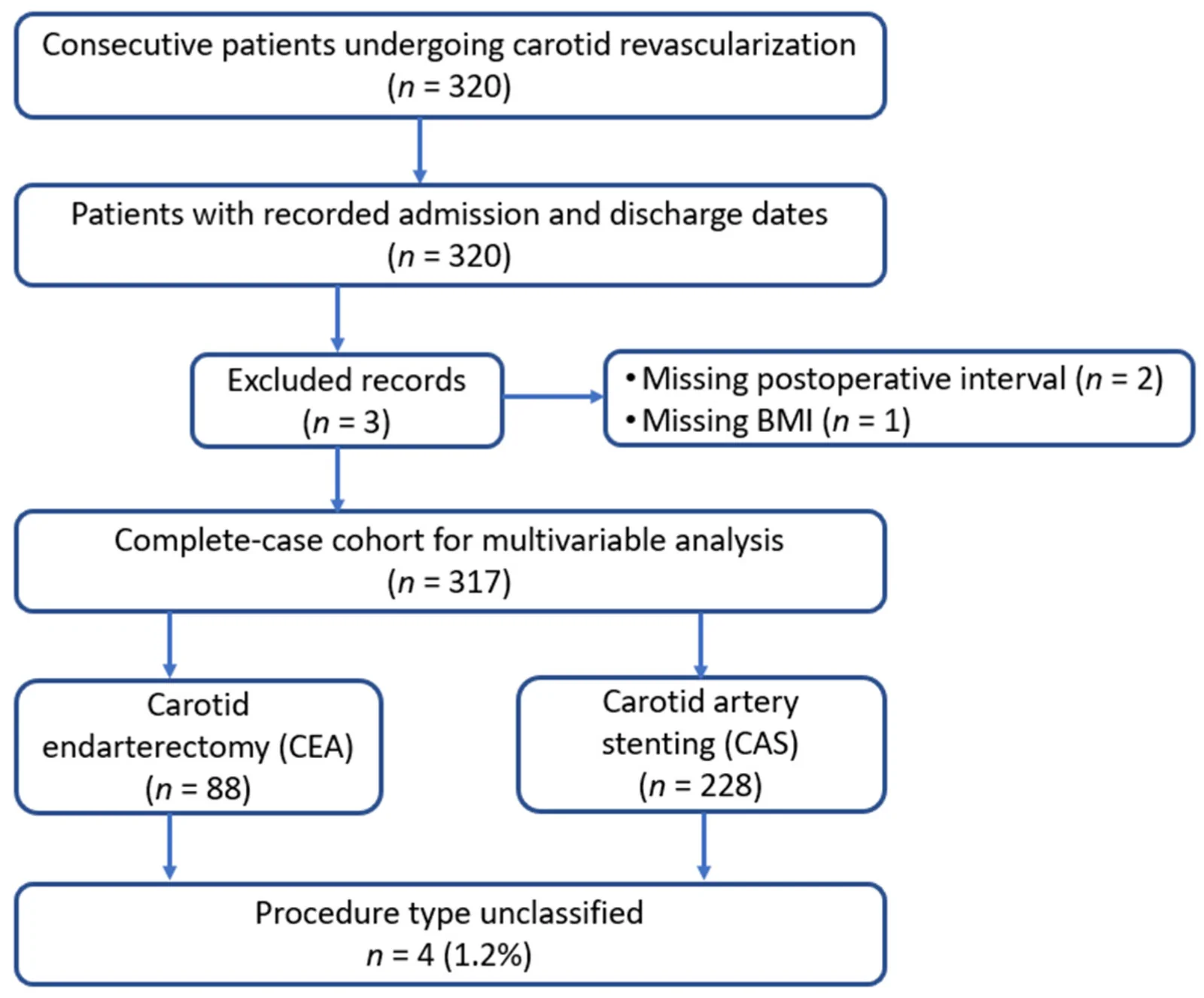
Patient flow. Of 320 consecutive patients treated between January 2018 and December 2025, 317 had complete data for the primary analysis; three were excluded (two without a derivable postoperative interval, one without body-mass index).

All patients in the registry who underwent carotid revascularization and had a recorded admission and discharge date were eligible; no age restriction was applied. The registry contained 320 consecutive patients, all of whom had admission and discharge dates and were included in the descriptive cohort. Carotid endarterectomy was performed in 88 patients (27.5%) and carotid artery stenting in 228 (71.2%); the procedure could not be classified in 4 patients (1.2%). Three patients were excluded from outcome modeling because a postoperative interval could not be derived (*n* = 2) or body-mass index was missing (*n* = 1), leaving 317 patients (72 with prolonged stay) for the primary analysis. The patient flow is summarized in Figure 1.

### 2.3. Outcome

The primary outcome was prolonged postoperative LOS, defined a priori as a postoperative stay exceeding the cohort 75th percentile. Postoperative LOS was calculated as total LOS minus preoperative LOS; the 75th percentile corresponded to 8 days, so patients staying more than 8 postoperative days were classified as having prolonged stay. A distribution-based threshold was chosen because no established clinical cutoff exists for postoperative LOS after carotid revascularization in a health system with this length of customary stay, and the commonly cited North American benchmark of >1–2 days reflects short-stay pathways that are not applicable here. To ensure that the findings did not depend on this dichotomization, postoperative LOS was also analyzed as a continuous count (negative binomial regression). Secondary in-hospital outcomes included a composite of any complication (wound infection, wound dehiscence, graft thrombosis, bleeding, postoperative transient ischemic attack, postoperative stroke, myocardial ischemia or myocardial infarction) and in-hospital death.

### 2.4. Variables and Definitions

Candidate baseline predictors were selected before modeling on the basis of clinical plausibility and prior literature, and were restricted to variables available at or before operation: age (modeled per 10-year increment), female sex, body-mass index (BMI), chronic heart failure, diabetes mellitus, post-infarction cardiosclerosis, grade 3 arterial hypertension, prior stroke, high-grade (>70%) carotid stenosis on duplex ultrasonography or angiography, and preoperative LOS. High-grade stenosis was coded positive when either imaging modality documented >70% narrowing. Most comorbidities were recorded in the source registry as presence flags, in which a positive entry indicated a documented diagnosis, and an absent entry was treated as absence of the recorded condition. Procedure type (carotid endarterectomy versus carotid artery stenting) and smoking status were recorded and examined in prespecified sensitivity analyses rather than in the primary model, to respect the events-per-variable constraint.

### 2.5. Statistical Analysis

Continuous variables are summarized as mean ± standard deviation and median with interquartile range (IQR), and categorical variables as counts and percentages. The association between baseline predictors and prolonged postoperative LOS was assessed using multivariable logistic regression, with results expressed as adjusted odds ratios (aORs) with 95% confidence intervals (CI). The predictor set was capped at ten preoperative variables to respect the events-per-variable constraint given 72 outcome events (events-per-variable ratio 7.2). Analyses were complete-case: of 320 patients, 317 had a derivable postoperative interval and complete predictor data and were included in the primary logistic model; missingness affected fewer than 2% of the cohort (two patients without a derivable postoperative interval and one without BMI). Model discrimination was assessed with the area under the receiver operating characteristic curve (AUC), calibration with the Hosmer–Lemeshow test and a calibration plot of observed versus predicted risk, and collinearity with variance inflation factors. Three sensitivity analyses were prespecified: (i) a negative binomial regression modeling postoperative LOS as a count (appropriate for the right-skewed, over-dispersed distribution); (ii) a logistic model additionally including procedure type (endarterectomy versus stenting); and (iii) a logistic model additionally including the in-hospital complication composite to quantify the contribution of perioperative events. Clinically plausible two-way interactions (age × sex, diabetes × high-grade stenosis, and procedure × high-grade stenosis) were explored and retained only if significant. Postoperative LOS was compared between patients with and without complications using the Mann–Whitney U test. A two-sided *p* < 0.05 defined statistical significance. Statistical analyses were performed using SAS OnDemand for Academics (version 3.81, Cary, NC, USA).

### 2.6. Ethics

The study protocol was approved by the Local Ethics Committee (LEC) of the Syzganov National Scientific Center of Surgery (Protocol No. 25, dated 6 April 2026). All procedures were conducted in accordance with the principles of the Declaration of Helsinki. Patient consent was waived due to the nature of the study, which involved the secondary analysis of deidentified data.

## 3. Results

### 3.1. Cohort Characteristics

The cohort comprised 320 patients (Table 1). Patients were predominantly male (239; 74.7%) and elderly, with a mean age of 71.1 ± 7.5 years (median 71, IQR 66–76). Mean BMI was 27.1 ± 4.1 kg/m^2^ and 76 patients (23.8%) were obese (BMI ≥ 30). The comorbidity burden was high: ischemic heart disease was documented in 294 patients (91.9%), chronic heart failure in 225 (70.3%), grade 3 hypertension in 181 (56.6%), post-infarction cardiosclerosis in 89 (27.8%) and diabetes mellitus in 87 (27.2%). Prior stroke was recorded in 72 patients (22.5%) and high-grade (>70%) stenosis in 220 (68.8%). Carotid endarterectomy was performed in 88 patients (27.5%) and carotid artery stenting in 228 (71.2%).

### 3.2. Length of Stay and In-Hospital Outcomes

**Figure 2:**
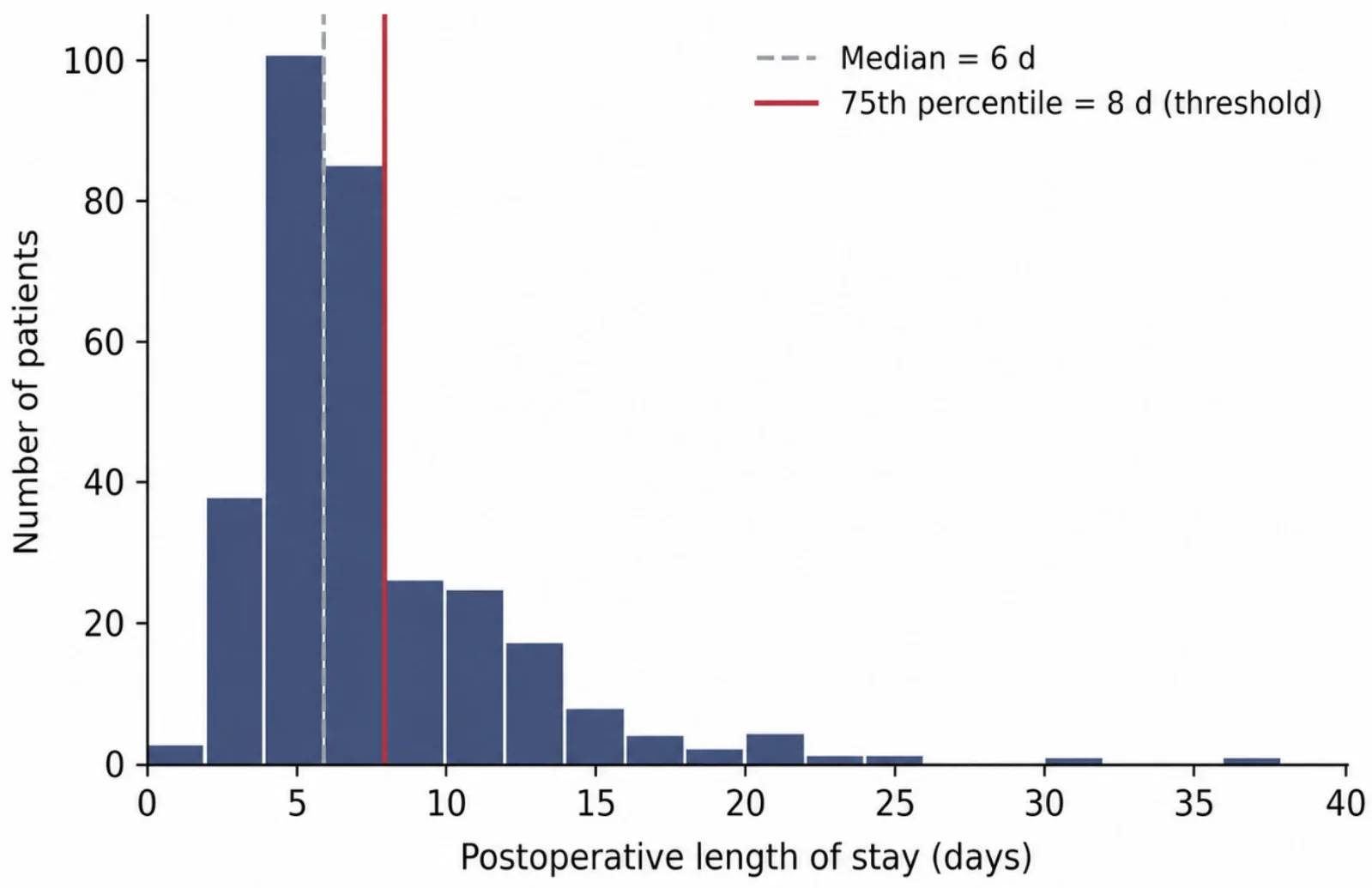
LOS distribution. Histogram of postoperative stay showing the right skew, the median (6 d) and the 75th-percentile threshold (8 d) that defines the outcome.

Median total LOS was 11 days (IQR 8–14; mean 12.4 ± 7.1). Median preoperative LOS was 4 days (IQR 2–7) and median postoperative LOS was 6 days (IQR 4–8; mean 7.1 ± 5.6). Postoperative stay was similar for the two procedures (endarterectomy: median 6 days, prolonged in 22.7%; stenting: median 5 days, prolonged in 21.9%). The discharge destination was not recorded. Applying the prespecified 75th-percentile threshold (>8 postoperative days), 72 patients (22.5%) had prolonged postoperative LOS (Figure 2). In-hospital complications were uncommon: any complication occurred in 11 patients (3.4%), comprising postoperative stroke in five (1.6%), myocardial infarction in two (0.6%), transient ischaemic attack in one (0.3%), bleeding in two (0.6%), graft thrombosis in one (0.3%) and wound infection in one (0.3%). Two patients (0.6%) died in hospital.

### 3.3. Predictors of Prolonged Postoperative Length of Stay

**Figure 3:**
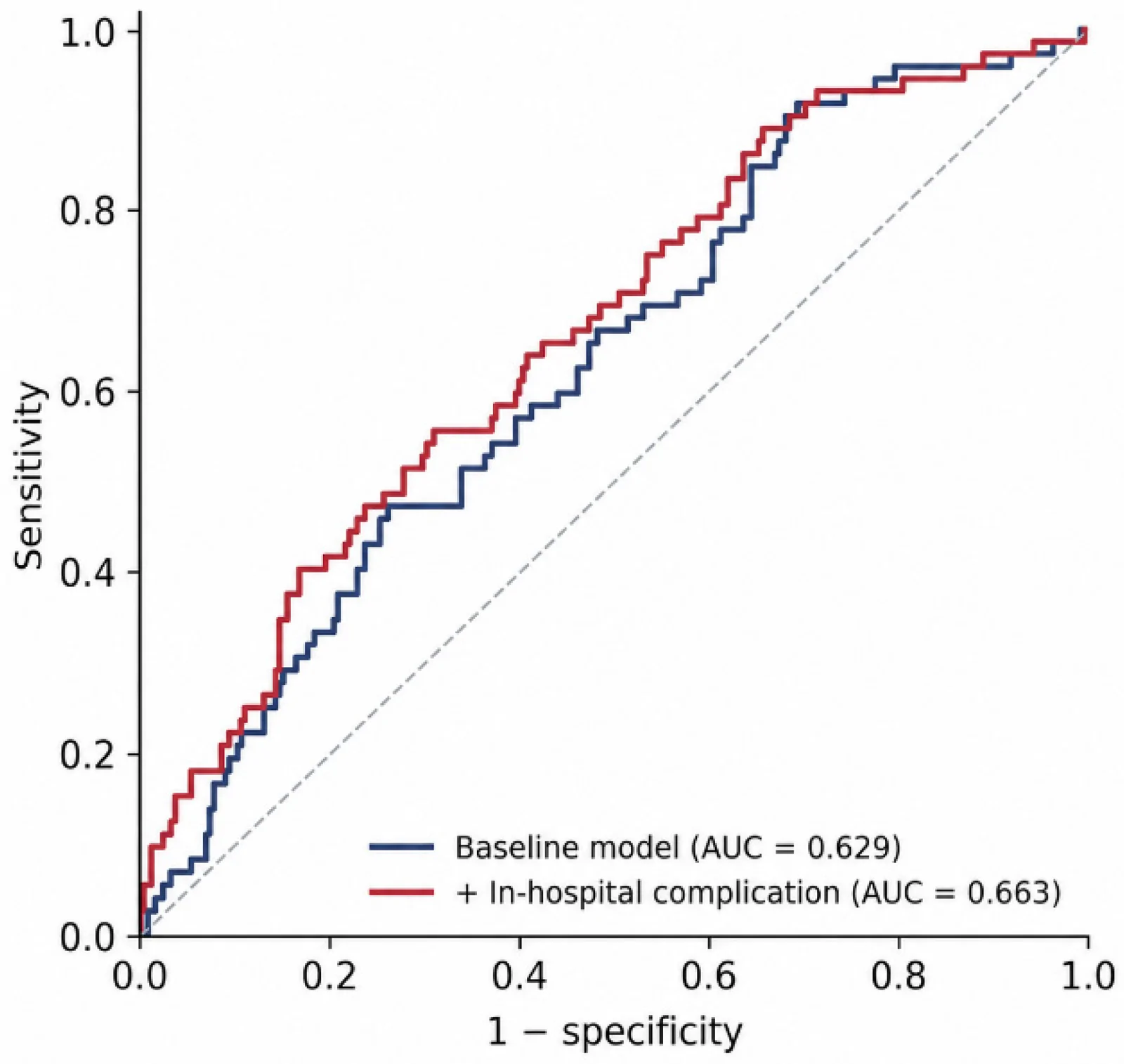
ROC curves. Baseline model (AUC 0.629) versus complication-adjusted model (AUC 0.663).

**Figure 4:**
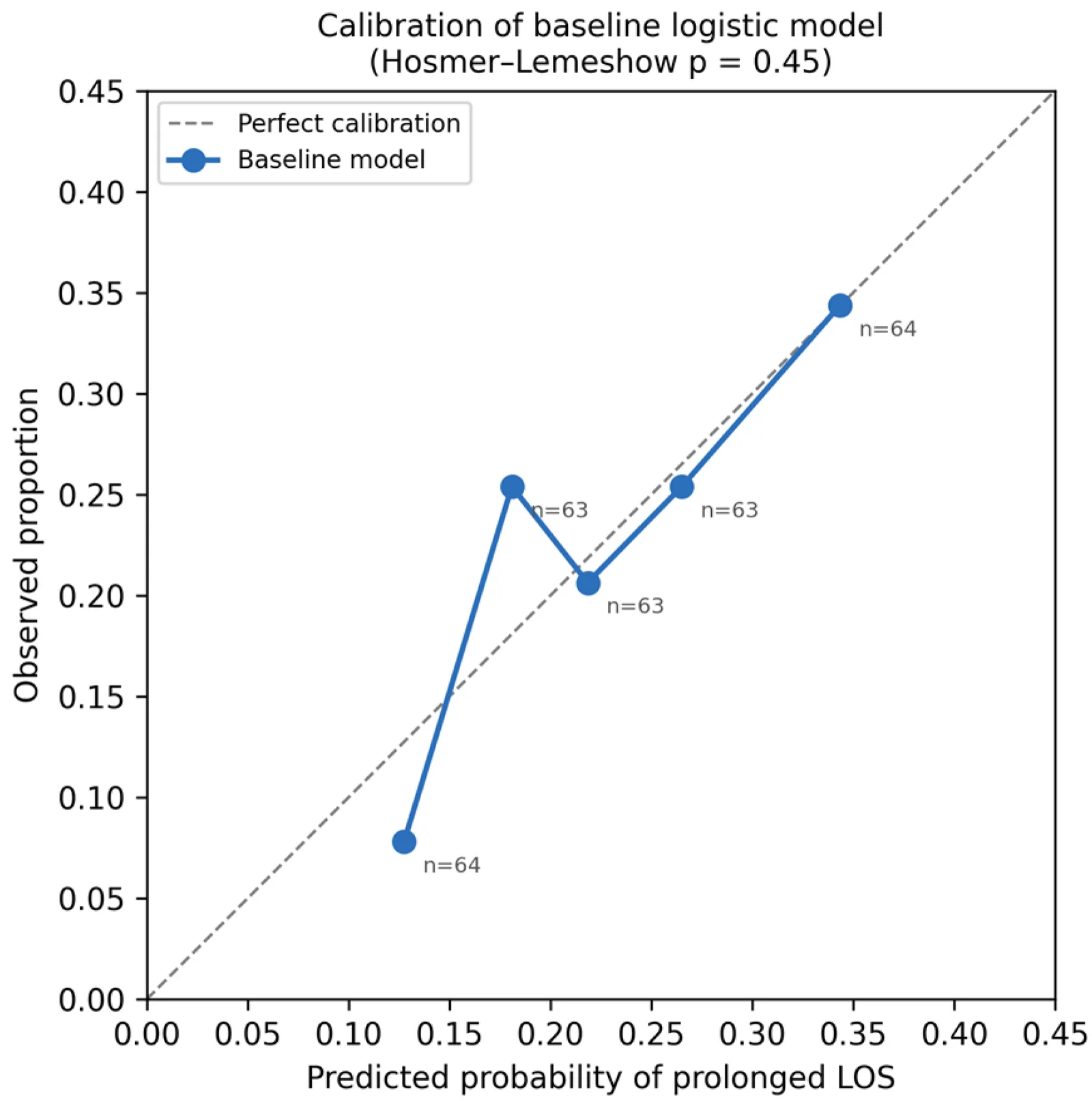
Calibration plot of the baseline logistic model, showing observed versus mean predicted probability of prolonged postoperative stay across risk quintiles (Hosmer–Lemeshow *p* = 0.45).

In the multivariable logistic regression (Table 2), no baseline characteristic was independently associated with prolonged postoperative LOS. High-grade stenosis showed the strongest, but non-significant, trend (aOR 1.76, 95% CI 0.93–3.33; *p* = 0.085). All other predictors—including age, sex, BMI, smoking, heart failure, diabetes, post-infarction cardiosclerosis, grade 3 hypertension, prior stroke and preoperative LOS—had confidence intervals spanning unity. The model discriminated poorly (AUC 0.629; pseudo-R^2^ 0.032) (Figure 3) and the overall likelihood-ratio test was non-significant (*p* = 0.36); calibration was adequate (Hosmer–Lemeshow *p* = 0.45; calibration plot, Figure 4) and no collinearity was detected. Adding procedure type did not change these findings: endarterectomy versus stenting was not associated with prolonged stay (aOR 1.14, 95% CI 0.61–2.14; *p* = 0.68) and left the other estimates essentially unchanged (AUC 0.634). No interaction term was significant. A negative binomial sensitivity analysis modeling postoperative LOS as a count broadly reproduced this pattern (Supplementary Table S1). Under a fixed-dispersion specification, no baseline predictor was significantly associated with length of stay.

### 3.4. In-Hospital Complications and Length of Stay

**Figure 5:**
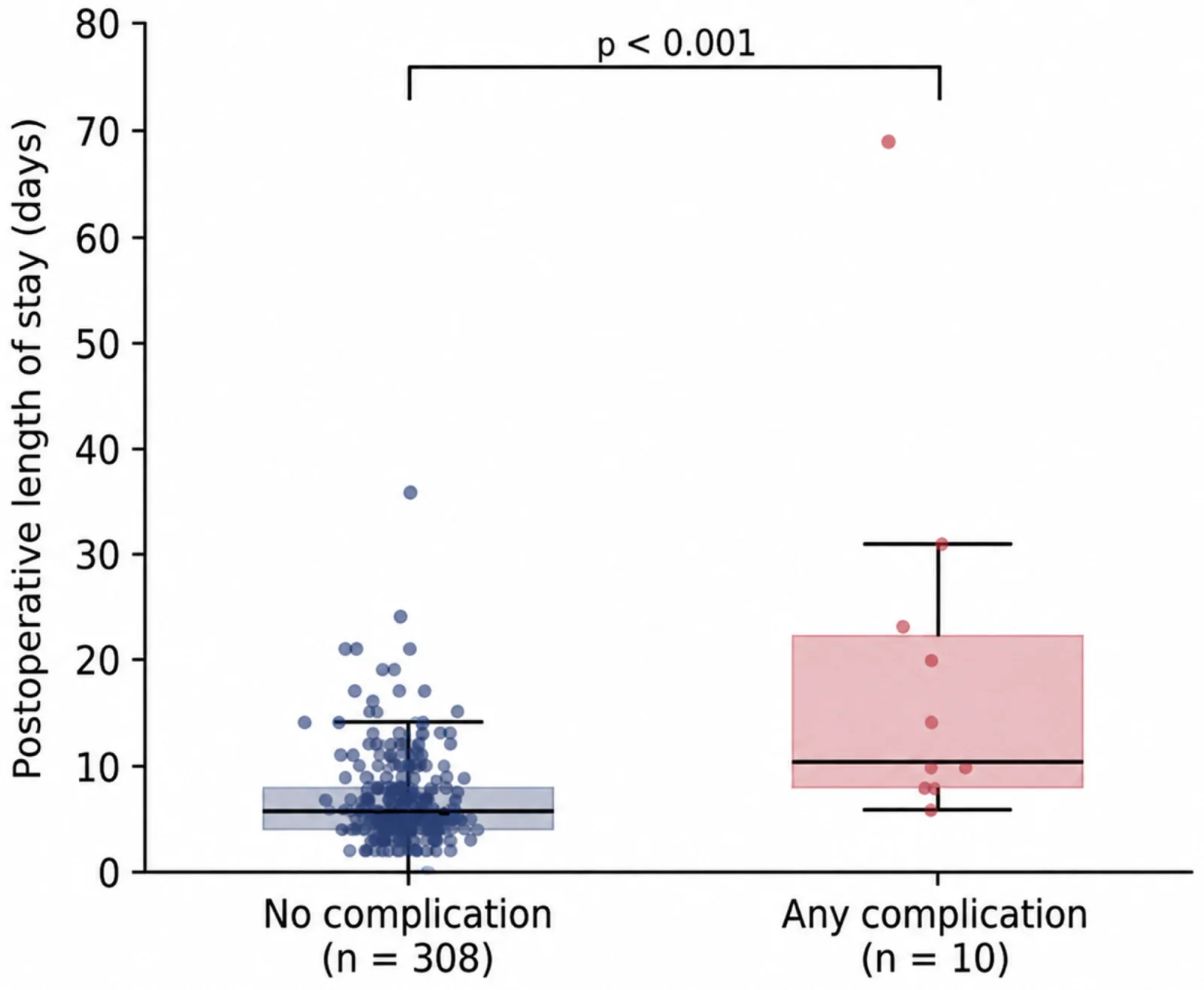
LOS by complication status. Box-and-jitter plot (median 6 (no complication) vs. 12.5 (any complication) days, *p* < 0.001), annotated with the adjusted odds ratio 8.50.

When the in-hospital complication composite was added to the model, it was the dominant correlate of prolonged stay (Table 3, Figure 5): patients who experienced any complication had more than eight-fold higher odds of prolonged postoperative LOS (aOR 8.50, 95% CI 2.04–35.40; *p* = 0.003). The confidence interval is wide, reflecting the small number of complication events (10 in the complete-case analytic sample; 11 in the full cohort), so the magnitude of this estimate should be interpreted cautiously. Model discrimination increased only modestly, from an AUC of 0.629 to 0.663, indicating that the added value of complications lies in their effect size rather than in improved preoperative discrimination. The effect on absolute stay was large: median postoperative LOS was 6 days (IQR 4–8) in patients without complications versus 12.5 days (IQR 8.5–22.3) in those with complications (*p* < 0.001). Prolonged stay occurred in 70.0% (7/10) of the complication patients for whom postoperative LOS was derivable, compared with 21.1% (65/308) of those without a complication. The length-of-stay comparison was therefore based on the 318 patients with a derivable postoperative interval (308 without and 10 with a complication), whereas the complication-adjusted logistic regression used the same 317 complete-case patients as the primary model, within which 10 experienced a complication; the single complication patient without a derivable postoperative interval was excluded from all length-of-stay analyses.

## 4. Discussion

In this elderly, comorbidity-heavy single-center carotid revascularization cohort from Kazakhstan, prolonged postoperative LOS could not be predicted from baseline patient characteristics. None of ten clinically rational preoperative variables reached independent significance, and the multivariable model discriminated only marginally better than chance (AUC 0.629). By contrast, the occurrence of an in-hospital complication—though rare at 3.4%—was associated with more than eight-fold higher odds of prolonged stay and more than doubled the median postoperative LOS. The central observation is therefore that, in this setting, variation in postoperative stay is associated more with what happens after the operation than with the case-mix presenting before it.

This pattern refines, rather than contradicts, the existing literature. Registry analyses from high-income systems have reported that age, symptomatic status, comorbidity and non-elective admission correlate with extended stay after carotid revascularization [8,9,15]. Those studies, however, draw on tens of thousands of patients with short median stays of one to two days, where even small absolute differences become statistically detectable and where early discharge is the norm. In a referral system with a median postoperative stay of six days and customary multi-day preoperative work-up, baseline risk is effectively diluted across a longer and more administratively determined hospitalization, leaving complications as the main source of clinically meaningful prolongation. Our complication finding is concordant with prior work in which perioperative adverse events are among the most consistent drivers of extended stay [9,16].

Two interpretive cautions follow. First, in-hospital complications occur after surgery and lie on the causal pathway between baseline risk and hospital stay; the model that includes them therefore illustrates mediation rather than identifying an independent baseline predictor, and it should not be read as a preoperative risk factor for prolonged stay. The distinction between prediction and causal inference matters here, because the practical implication—preventing complications—differs fundamentally from preoperative risk stratification. Second, the poor discrimination of the baseline model (AUC 0.629) does not by itself establish that preoperative characteristics are unimportant. It is at least as plausible that the predictors most relevant to LOS in this setting—symptomatic status, urgency, anesthetic and operative details, and postoperative blood-pressure management—were unavailable, that presence-flag coding introduced measurement error that diluted associations toward the null, and that postoperative LOS, being partly determined by non-clinical discharge logistics, is intrinsically difficult to predict from preoperative data alone.

The absence of a baseline predictive signal should be interpreted with the study’s limited power in mind. With only 72 outcome events and 11 complications, the study was underpowered to detect modest associations, and the uniformly non-significant baseline predictors are as consistent with type II error as with a true absence of effect. With that caveat, the findings suggest that, in comparable health systems, the baseline variables available in this registry offered limited ability to identify patients at risk of prolonged stay. Because several potentially important preoperative and perioperative variables were not captured, this most likely reflects the limited predictive performance of the available data rather than evidence that preoperative risk stratification is inherently unhelpful; attention may nonetheless be usefully directed toward complication prevention and early recognition. The near-significant association of high-grade stenosis with longer stay is hypothesis-generating and may reflect more complex disease or more cautious postoperative observation, but it should not be over-interpreted given the wide confidence interval.

Because complications, rather than baseline characteristics, were the dominant correlate of prolonged stay in this elderly, comorbidity-heavy cohort, individualized peri-procedural risk reduction is a biologically plausible lever for shortening hospitalization. External evidence supports the feasibility of this approach: in the TARGET-CAS study of 1176 carotid artery stenting procedures, selecting the embolic-protection device and stent type for each patient on the basis of a thorough non-invasive work-up (the tailored-CAS algorithm) achieved a 30-day death, stroke or myocardial-infarction rate of 2.38%, with advanced age identified as a predictor of peri-procedural death [17]. In a referral population as old and comorbid as ours, in which roughly seven of ten patients were treated by stenting, such patient-level tailoring of technique and of post-procedural surveillance offers a concrete mechanism by which complication prevention—rather than preoperative risk stratification—could translate into shorter stay. We emphasize, however, that our data are associational and cannot test this hypothesis directly; the tailored-CAS experience is cited as external proof of concept and a rationale for prospective evaluation, not as evidence generated by the present cohort.

Strengths and limitations: Strengths include a complete consecutive cohort with a prespecified outcome definition and predictor set, adherence to STROBE, and prespecified sensitivity analyses—including models for procedure type, a continuous LOS outcome and complications—that converged on the same conclusion. The study also addresses a genuine geographic gap, providing what is to our knowledge the first such analysis from Central Asia. Several limitations temper interpretation. First, the single-center, retrospective design limits generalizability and precludes causal inference. Second, most comorbidities were recorded as presence flags, so an absent entry cannot be distinguished from an undocumented condition; this non-differential misclassification would tend to bias associations toward the null and may partly explain the uniformly non-significant baseline predictors. Third, several variables known to influence postoperative stay were unavailable, including symptomatic versus asymptomatic presentation, urgency of surgery, anesthesia type, operative and clamp times, intensive-care admission, surgeon experience and postoperative blood-pressure management; residual confounding from these factors is therefore likely. Although procedure type was available and was not associated with prolonged stay, the indication for intervention (symptomatic versus asymptomatic stenosis) was not consistently recorded and could not be analyzed. Fourth, the small number of complications (*n* = 11) yields a wide confidence interval around the dominant effect estimate and precludes modeling of individual complication types. Finally, the events-per-variable ratio of 7.2 is below the conventional target of 10, so the baseline model carries some risk of overfitting and should be read cautiously; the concordance of the independent sensitivity analyses provides partial reassurance.

Because postoperative LOS in this cohort is considerably longer than in Western series—largely a function of how care is organized and how discharge is decided rather than of clinical severity—the specific determinants identified here may not generalize beyond similar health systems. Future work should validate these findings in multi-center Central Asian cohorts with prospectively captured, fully coded comorbidity and procedural data, and should test whether structured complication-prevention pathways shorten stay more effectively than preoperative risk stratification. Linking LOS to downstream cost and to longer-term stroke outcomes would further clarify its value as a quality metric in this region.

## 5. Conclusions

Prolonged postoperative LOS after carotid revascularization in this Kazakhstani cohort was not explained by baseline case-mix but was associated with in-hospital complications, which lie on the causal pathway to prolonged stay. LOS in this setting may be better understood as a marker of perioperative events than of preoperative risk. These observational findings are hypothesis-generating: they suggest that complication prevention is a promising lever for shortening stay, but this should be tested prospectively rather than assumed. With only 11 complications, the present study can establish an association between complications and prolonged stay but cannot identify which specific preventive strategy would reduce hospitalization; the tailored-CAS experience is therefore cited only as external proof of concept and a rationale for prospective evaluation, not as evidence generated by the present cohort.

## Figures and Tables

**Table 1:** Baseline characteristics of the cohort (N = 320).

Characteristic	Value
Age, years—mean ± SD	71.1 ± 7.5
Age, years—median (IQR)	71 (66–76)
Male sex—*n* (%)	239 (74.7)
Female sex—*n* (%)	81 (25.3)
Body-mass index, kg/m^2^—mean ± SD	27.1 ± 4.1
Obesity (BMI ≥ 30)—*n* (%)	76 (23.8)
Ischemic heart disease—*n* (%)	294 (91.9)
Post-infarction cardiosclerosis—*n* (%)	89 (27.8)
Chronic heart failure—*n* (%)	225 (70.3)
Diabetes mellitus—*n* (%)	87 (27.2)
Atrial fibrillation—*n* (%)	19 (5.9)
Grade 3 arterial hypertension—*n* (%)	181 (56.6)
Prior stroke—*n* (%)	72 (22.5)
Encephalopathy—*n* (%)	71 (22.2)
Smoking—*n* (%)	112 (35.0)
High-grade stenosis > 70%—*n* (%)	220 (68.8)
Carotid endarterectomy—*n* (%)	88 (27.5)
Carotid artery stenting—*n* (%)	228 (71.2)

SD, standard deviation; IQR, interquartile range; BMI, body-mass index.

**Table 2:** Multivariable logistic regression for prolonged postoperative length of stay (>8 days).

Predictor	Adjusted OR (95% CI)	*p* Value
Age (per 10 years)	0.84 (0.58–1.22)	0.357
Female sex	0.56 (0.28–1.14)	0.111
Body-mass index (per unit)	1.02 (0.95–1.09)	0.616
Smoking	1.04 (0.57–1.91)	0.901
Chronic heart failure	0.96 (0.49–1.88)	0.905
Diabetes mellitus	1.41 (0.77–2.58)	0.261
Post-infarction cardiosclerosis	1.25 (0.68–2.32)	0.471
Grade 3 hypertension	0.69 (0.38–1.25)	0.218
Prior stroke	1.21 (0.64–2.29)	0.554
High-grade stenosis >70%	1.76 (0.93–3.33)	0.085
Preoperative LOS (per day)	1.00 (0.93–1.06)	0.899

OR, odds ratio; CI, confidence interval; LOS, length of stay. Model: N = 317, 72 events; AUC 0.629; Hosmer–Lemeshow *p* = 0.45.

**Table 3:** Postoperative length of stay by occurrence of in-hospital complication.

	No complication (*n* = 308)	Any complication (*n* = 10)
Postoperative LOS, days—median (IQR)	6 (4–8)	12.5 (8.5–22.3)
Prolonged postoperative LOS—*n* (%)	65 (21.1)	7 (70.0)

LOS, length of stay; IQR, interquartile range; cell counts are based on the 318 patients with a derivable postoperative interval (308 without and 10 with a complication); two patients (one in each group) lacked a derivable postoperative interval and were excluded from this table. Between-group comparison of postoperative LOS *p* < 0.001 (Mann–Whitney U); adjusted odds ratio for prolonged stay given any complication 8.50 (95% CI 2.04–35.40).

## Data Availability

The data presented in this study are available from the corresponding author upon reasonable request due to the data protection restrictions of the clinic.

## Supplementary Materials

The following supporting information can be downloaded at: https://www.mdpi.com/article/10.3390/jcm15176548/s1, Table S1: Negative binomial regression for postoperative length of stay modeled as a count (N = 317). Incidence rate ratios (IRRs) are shown for the maximum-likelihood dispersion model; two-sided *p*-values are given for the fixed-dispersion (α = 1.0).

## Abbreviations

The following abbreviations are used in this manuscript:

LOS	length of stay
STROBE	Strengthening the Reporting of Observational Studies in Epidemiology
BMI	body-mass index
IQR	interquartile range
AUC	area under the receiver operating characteristic curve
CEA	carotid endarterectomy
CAS	carotid artery stenting
OR	odds ratio
aOR	adjusted odds ratio
CI	confidence interval
SD	standard deviation
