# Supplementary material for: Determinants of Prolonged Postoperative Length of Stay After Carotid Revascularization: A Single-Center Registry Analysis from Kazakhstan
Source: J Clin Med. 2026 Aug 25;15(17):6548. doi: 10.3390/jcm15176548 (PMC13566745; doi:10.3390/jcm15176548)
Supplement: Supplementary file 1 [file jcm-15-06548-s001.zip › jcm-4496400-supplementary.pdf]

Supplementary Material

Table S1. Negative binomial regression for postoperative length of stay modelled as a count (N = 317). Incidence rate ratios (IRRs) are shown for the maximum-likelihood dispersion model; two-sided p-values are given for the fixed-dispersion (α = 1.0).

Predictor	IRR (95% CI) — α estimated	p
Age (per 10 years)	0.95 (0.86–1.04)	0.519
Female sex	0.87 (0.74–1.02)	0.344
BMI (per unit)	1.00 (0.99–1. 02)	0.913
Chronic heart failure	0.96 (0.82–1.13)	0.824
Diabetes mellitus	1.12 (0.96–1.31)	0.372
Post-infarction cardiosclerosis	1.07 (0.91–1.24)	0.654
Grade 3 hypertension	0.81 (0.70–0.94)	0.111
Prior stroke	1.13 (0.97–1.33)	0.402
High-grade stenosis >70%	1.16 (1.00–1.35)	0.286
Preoperative LOS (per day)	1.00 (0.98–1.01)	0.852

IRR, incidence rate ratio; CI, confidence interval; LOS, length of stay. Under the dispersion model no predictor was significant.
